# Improvement of Formability in Parallel Double-Branched Tube Hydroforming Combined with Pre-Forming and Crushing Processes

**DOI:** 10.3390/ma17061327

**Published:** 2024-03-13

**Authors:** Mingtao Chen, Jinhao Hu, Yunya Xiao, Junwei Liang, Zhiwei Ye, Hongchao Wu, Feng Zhou, Guisheng Mao, Hui Long, Wei Tang, Xiaoting Xiao

**Affiliations:** 1 School of Intelligent Engineering, Shaoguan University, Shaoguan 512005, China; cmt9090@163.com (M.C.); 13750101213@163.com (J.H.); 13826379720@139.com (Y.X.); 13682404656@163.com (J.L.); 13553984360@163.com (Z.Y.); 19830288541@163.com (H.W.); 15602346607@163.com (G.M.); longhui32603260@126.com (H.L.); tangweijmsdx@163.com (W.T.); 2 School of Materials and Energy, Guangdong University of Technology, Guangzhou 510006, China; xxting1@163.com

**Keywords:** parallel double-branched tube, crushing, tube hydroforming

## Abstract

In hydroforming of parallel double-branch tubes, the material entering the branch zone is obstructed by material accumulation in the main tubes and corners, which decreases the branch height. A tube hydroforming approach is combined with pre-forming and crushing (THPC) to mitigate this problem. A larger diameter tube blank is flattened for pre-forming and then subjected to radial compression for crushing. In the next step, hydroforming forms the parallel double-branch tubes. Experiments and numerical simulations are then carried out to analyze the effect of traditional tube hydroforming (TTH) and the proposed THPC process on the formability of parallel double-branch tubes. The results show that for tubes obtained via THPC, the tube burst pressure increases by 27.5% and the branch height increases 2.37-fold compared to TTH. Additionally, the flattening, pre-forming, and crushing stages cause work hardening of the tube when using the TPHC process. Flattened tubes undergo radial compression to improve the material flowing into the branch tube. The formability of parallel double-branched tubes can be improved by using the TPHC process. Consequently, tube hydroforming, combined with pre-forming and crushing, has been confirmed as a feasible forming process for fabricating parallel double-branch tubes.

## 1. Introduction

Recently, tube hydroforming has been extensively employed in fabricating lightweight tube fittings such as exhaust parts, bathroom faucet spouts, front and rear axles, and steel panic bars [1,2]. Tube hydroforming has several advantages over traditional forming techniques such as stamping and welding. It requires fewer manufacturing steps, has no welding seams, and results in lighter products with higher strength and rigidity [3,4].

Multi-way tubes are widely used in ship and automobile piping systems [5]. The tube hydroforming process is commonly used to manufacture multi-way tubes. Hence, many studies on the subject are available, examining the deformation behavior during the forming process of multi-way tubes. Cui et al. [6,7] proposed a novel multi-step hydroforming technique for producing complex T-shaped tubular components made of nickel-based superalloys. The wall thickness and stress states were analyzed during the tube-forming process—the tube material accumulated in the transition zone due to axial feeding, continuously increasing wall thickness. Arman et al. [8] utilized a low-velocity impact hydroforming process to produce seamless T-shaped tubes. The appropriate impact energy and fluid volume were crucial in producing flawless components. Sornin et al. [9] investigated plastic instability analysis using the Modified Maximum Force Criterion. They proposed a continuum damage model that accounted for the pressure sign and the stress triaxiality threshold during the T-shaped tube hydroforming process. Furthermore, Vu et al. [10] examined the influence of internal pressure, axial force, counterforce, axial displacement, and friction on protrusion height during Y-shaped tube hydroforming. Jirathearanat et al. [11] investigated the effect of process parameters on the formability of the protrusion during Y-shaped tube hydroforming. As the length of the tube decreased, the height of the protrusion formed increased. Chen et al. [12] investigated the deformation behavior, microstructure, and mechanical properties of the parallel double-branch tubes during tube hydroforming. It was found that the wall thickness increased in the main and corner zones, while it decreased in the branching zone. Finally, Hajime et al. [13] investigated the effect of lubrication and tube length on the forming defects and the forming limit in the T-shape microtube hydroforming. The flow of the microtube material into the die cavity was restricted due to the increase in frictional resistance with longer tube lengths.

Additionally, the forming parameters of the multi-way tube hydroforming process have been optimized to improve the process outcomes. Moataz et al. [14] proposed an adaptive heuristic nonlinear mathematical model to optimize the loading path during T-shaped tube hydroforming. The internal pressure and axial load were adaptively minimized by utilizing machine learning techniques. Fethi et al. [15] proposed using an artificial neural network modeling to estimate the parameters of T-shaped tube hydroforming. The material behavior was described via the micromechanical Gurson–Tvergaard–Needleman damage model. Ali et al. [16] used a multi-step forming approach to manufacture five-branched stainless steel tubes of different diameters. The optimal forming parameters were found through response surface optimization. Mehran et al. [17] applied an annealing algorithm to obtain the optimal load parameters of the X- and Y-shaped tubes. The effectiveness of the optimization method was experimentally confirmed. Mohamed et al. [18] proposed an explicit dynamic approach and an automatic surrogate model to predict an acceptable T-shaped tube with minimum wall thickness variations during tube hydroforming. The best tool dimensions and ideal punch stroke were obtained through the proposed approach. Kadkhodayan et al. [19] proposed the insertion of mathematical models into an evolutionary algorithm in order to design the optimal load paths for T-shaped tube hydroforming. The optimal load paths were instrumental in improving the thickness distribution in the part. Brooghani et al. [20] obtained the optimal loading path for producing a T-shaped tube using the multilevel-response surface method. The findings indicated a marked improvement in the thickness variation.

Studies have been carried out to examine the deformation behavior and optimize the forming parameters. The formability of multi-way tubes was enhanced by improving the material flow during tube hydroforming. The accumulation of material increased the wall thickness of the main tube and the corner zones. The accumulation resulted from the high-friction forces occurring when applying axial feeding during the hydroforming of multi-way tubes. The wall thickness of the branch tube area was reduced due to the increase in internal pressure. In practical production, after the multi-way tube was formed via the tube hydroforming process, the overly thickened areas were machined by turning to meet the usage requirements. In that case, the number of processing steps and the production costs increased.

**Figure 1:**
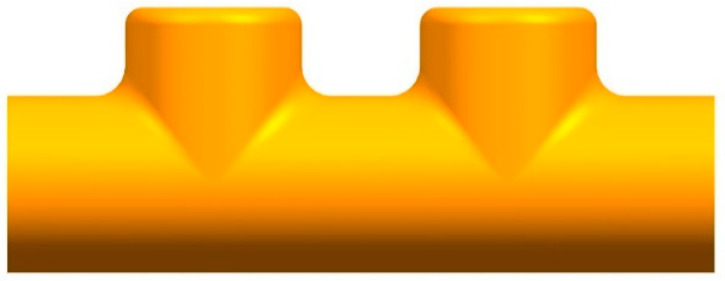
A schematic of a parallel double-branch tube.

The T-shaped and Y-shaped tubes have a single branch, meaning that the branch can be replaced via axial feeding during tube hydroforming. The high branch can be obtained via a suitable relationship between axial feeding and internal pressure. Compared to T-shaped and Y-shaped tubes, the parallel double-branch tube (see Figure 1) has two branches, meaning that the outer side of each branch can only be replaced via axial feeding. Consequently, the branches are asymmetric, and their height is reduced. Additionally, if the parallel double-branch tubes are too long, the material flow is hindered due to increased frictional resistance.

Researchers have proposed several methods to address material flow difficulties and improve the formability of tubes with irregular cross-sections and excessively long structures. Morphy et al. [21] proposed a pressure sequence hydroforming process. Lower pressure was applied to the tube once the die was closed, enabling the material to flow into the mold cavity more smoothly due to reduced friction. When the die was closed, the hydraulic pressure was increased to form the tube component. Next, Yang et al. [22] investigated the effect of a pulsating loading path on the deformation behavior of tube components during tube hydroforming with radial crushing. The authors determined that the formability of tubes was improved. Hwang et al. [23,24] investigated the deformation behavior of triangular and rectangular cross-section tube components produced using the hydroforming process with crushing. It was found that it was possible to significantly decrease the maximum internal pressure and crushing force needed in the crushing process. Nikhare et al. [25,26] investigated the internal pressure and die closing force required during low-pressure tube hydroforming (LPTH) in a square cross-section geometry and compared the results with those obtained via high-pressure tube hydroforming (HPTH). During LPTH, the internal pressure and die closing force needed were only 6.5% and 57.5%, respectively, of what was required in the HPTH process. Furthermore, the HPTH process was affected by friction, while friction was not significant in the LPTH process. Therefore, combining hydroforming and crushing processes has become an important technology for fabricating tube components with various cross-sections.

However, compared to tube hydroforming, available studies on hydroforming and crushing in the manufacturing of multi-way tubes are too narrow to provide the knowledge needed to design the process efficiently. Therefore, a novel THPC process for manufacturing parallel double-branch tubes was proposed. Firstly, the basic principles of the THPC process were given. The forming process was then presented, and a comparison and analysis of the branch height and wall thickness during both THPC and TTH processes was carried out through a combination of finite element analysis and experimental methods. Lastly, the changes in stress at the significant feature positions were examined.

## 2. THPC Process—Key Principles

**Figure 2:**
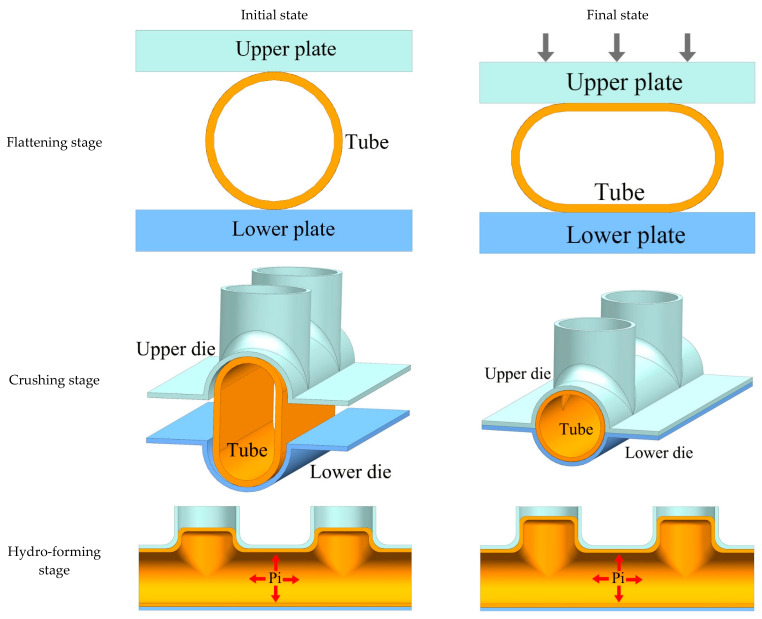
Schematic diagram of the THPC process of parallel double-branch tubes (Pi is the internal pressure. The red arrows indicate internal pressure acting in any direction within the tube.).

The schematic diagram of the THPC process for the production of parallel double-branch tubes is shown in Figure 2. The process comprises three stages: workpiece flattening for pre-forming, crushing, and hydroforming. The initial circular tube blank is placed between the upper and lower plates in the flattening stage. The tube blank is flattened by applying downward pressure on the upper plate. The initial circular tube blank has a larger diameter than the main tube die cavity, providing additional material for forming the branching zone. The flattened tube is placed into the forming die in the crushing stage. The upper die’s downward force shapes the tube without applying internal pressure. Lastly, the internal pressure gradually increases in the hydroforming stage until the formation is completed.

## 3. Tubular Components and Methods

### 3.1. Geometric Dimensions of the Parallel Double-Branch Tube

**Figure 3:**
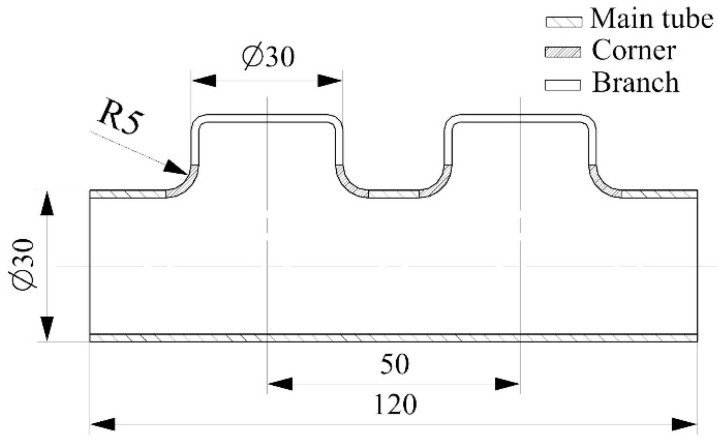
Segment of the parallel double-branch tube with dimensions.

Figure 3 shows the geometric dimensions of the parallel double-branch tube specified in this paper. The parallel double-branch tube comprised a main tube, a branch tube, and a corner. Main and branch tubes had 30 mm diameters and were 120 mm long. The center distance between branch tubes was 50 mm, with a corner radius of 5 mm between the branch tube and the main tube.

### 3.2. Material

**Figure 4:**
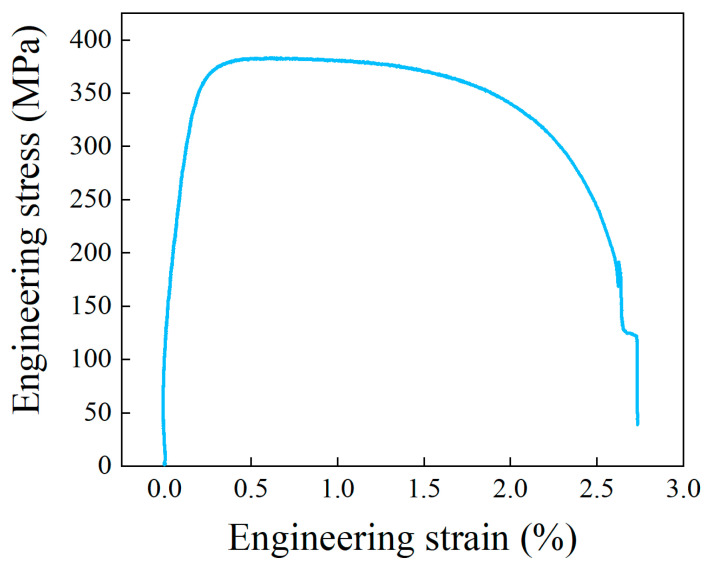
Engineering stress–strain curve of seamless copper tube.

The as-received tube materials were used to form the parallel double-branch tube. A commercial pure copper tube with a purity of 99.9% was utilized. Seamless pure copper tubes were manufactured using an extrusion process. Furthermore, the mechanical properties of the copper tube in the axial direction were tested via a uniaxial tensile test on the Instron 5582 machine. Figure 4 illustrates the engineering stress–strain curve of the copper tube. The copper tube’s yield and ultimate tensile strength were 378.7 MPa and 383.4 MPa, respectively. The extrusion-hardened copper tube exhibited a low degree of plasticity, with an elongation rate of 2.73%.

### 3.3. Experimental Setup

#### 3.3.1. Flattening to Pre-Form

**Figure 5:**
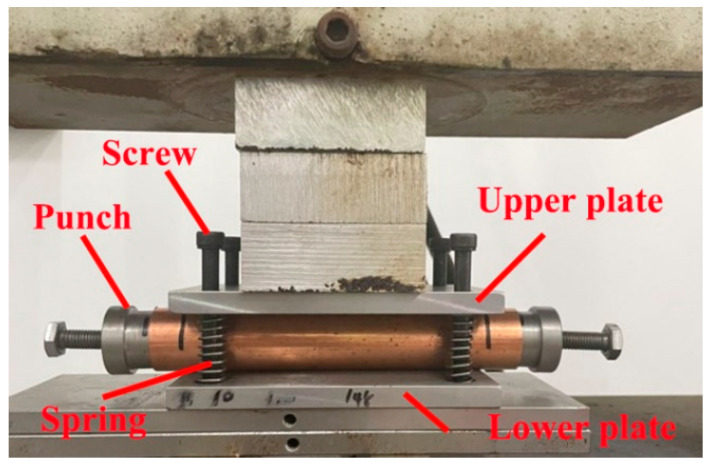
Experimental setup for flattening.

The device shown in Figure 5, comprising an upper plate, a lower plate, screws, springs, and punches, was used to flatten the tube blanks. A punch was inserted into the tube blank to protect the tube end from distortion. The tube blank was placed between the upper and lower plates and compressed by a universal testing machine with a maximum load of 30 t. The amount of compression was digitally controlled.

#### 3.3.2. Experimental Setup for Crushing and Hydroforming

**Figure 6:**
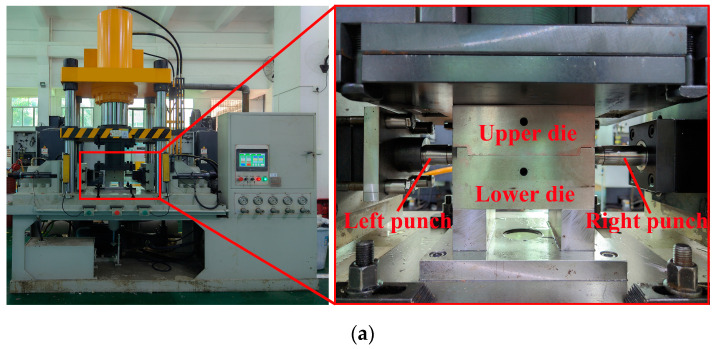

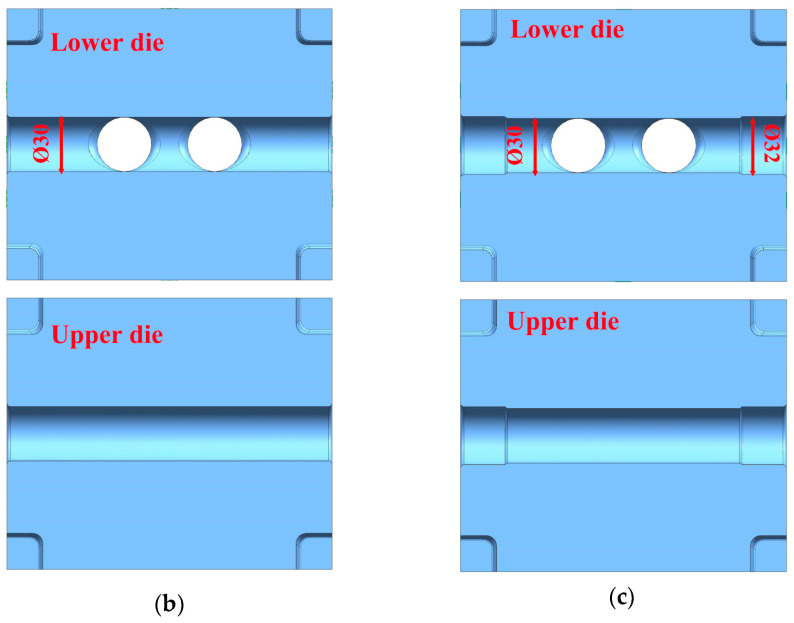
Experimental setup for crushing and hydroforming: (**a**) experimental setup, (**b**) die for the TTH process, and (**c**) die for the THPC process.

The equipment for crushing and hydroforming (Figure 6) includes an internal high-pressure forming machine and a forming die. The forming die comprises upper and lower dies and left and right punches. The internal high-pressure forming machine can exert a clamping force of 120 t and a hydraulic bulging force of 120 MPa. The tube blank was placed into a die, and the upper die was then pressed downward to complete the crushing. The axial feeding length for the sealing was only 1.5 mm. Finally, the tube-forming process was concluded by gradually increasing the internal pressure. 

### 3.4. Experimental Scheme

The effects of TTH and THPC processes on the formability of a parallel double-branch tube are investigated in this section. Table 1 shows the experimental schemes of both forming processes. For the TTH, a tube blank with a diameter of 30 mm and a length of 180 mm was used. The internal pressure was gradually raised until the tube ruptured. For the THPC, a tube blank with a diameter of 32 mm (a larger diameter was needed to provide additional material) and a length of 180 mm was used. The circumference of a tube blank with a diameter of 32 mm was 100.5 mm. This circumference was larger than that of a tube blank with a diameter of 30 mm, which was 94.2 mm. The tube blank was first flattened. The amount of flattening was 13 mm, while the length of the flattening was 132 mm. Then, the tube blank was crushed without the application of internal pressure. Finally, the internal pressure gradually increased until the tube ruptured.

### 3.5. Finite Element Model

**Figure 7:**
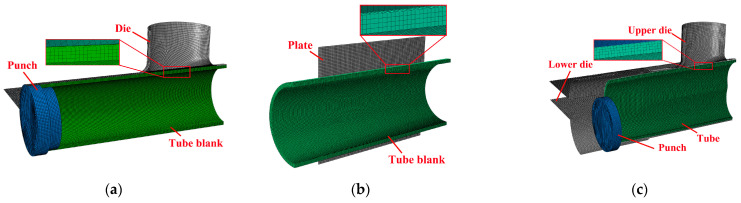
Finite element model: (**a**) TTH process model, (**b**) flattening model, and (**c**) crushing and hydroforming models.

A finite element analysis (FEA) model was established using ABAQUS v6.13-1/Explicit software, enabling the analysis of the deformation process, stresses, and strains, as illustrated in Figure 7. The tube blank was meshed using solid elements C3D8R with an approximate mesh size of 0.4 mm. The die, punch, and flattening plate were assigned as discrete rigid shell elements R3D4. A predefined field was set in the crushing and hydroforming stages. The tube blank at the end of flattening was taken as the initial tube blank for the crushing and hydroforming stages, while the stress and strain fields were retained. The friction tests were carried out using several lubricants, and the friction coefficients during hydroforming ranged from 0.03 to 0.09 [27]. For the tube/punches and tube/die interfaces, a value of 0.05 was used as the elastic Coulomb friction coefficient.

## 4. Results and Discussion

### 4.1. Experimental Results

**Figure 8:**
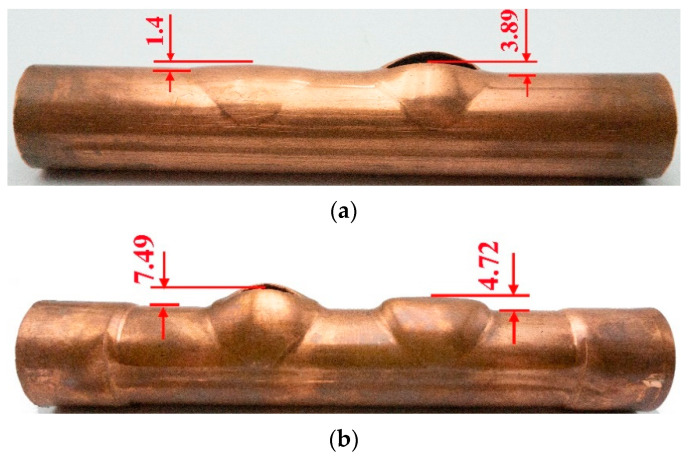
Experimental result for final forming parts: (**a**) TTH and (**b**) TPHC processes.

Figure 8 shows the parallel double-branch tubes obtained from the experiment using the specimen obtained through TTH and THPC processes. When the internal pressure was raised to 40 MPa (TTH) and 51 MPa (THPC), bursting occurred at one of the branches of the tube part. The reason for such behavior might be the non-uniform deformation during the forming stage of the two processes. The ruptured internal pressure increased by 27.5% for the hydroforming using the THPC compared to the TTH process. This phenomenon could be due to material hardening after the flattening and crushing stages.

**Figure 9:**
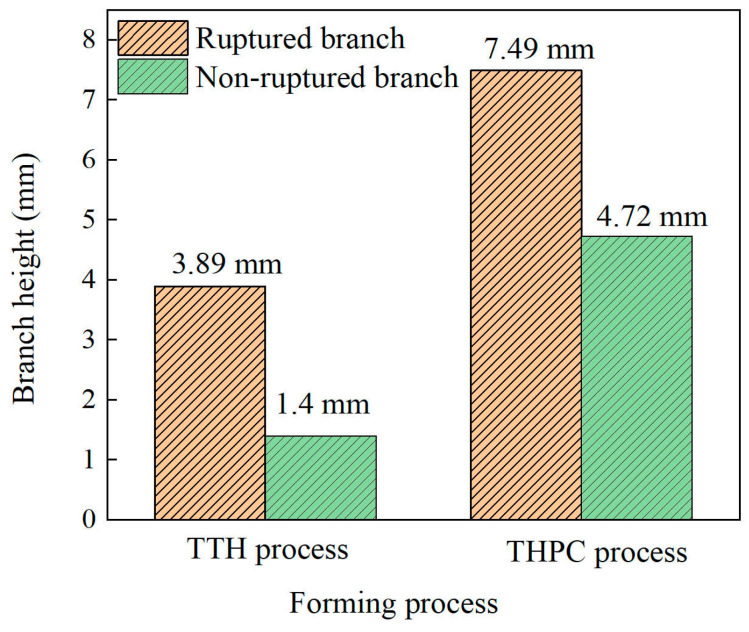
Branch heights achieved through the TTH and THPC process.

Figure 9 shows the branch heights achieved through both forming processes. The branch height was determined by measuring the distance from the outside of the main tube to the top of the branch (Figure 8). Using the TTH process, the branch heights for the non-ruptured and ruptured branches were 1.4 mm and 3.89 mm, respectively. In comparison, the THPC process yielded branch heights of 4.72 mm for the non-ruptured and 7.49 mm for the ruptured tubes. Compared with the TTH process, the branch height increased significantly (2.37-fold) when using the THPC process. Consequently, the branch height was increased, and the tube formability was significantly improved compared to the TTH process.

### 4.2. Thickness Distribution and Deformation Behavior

**Figure 10:**
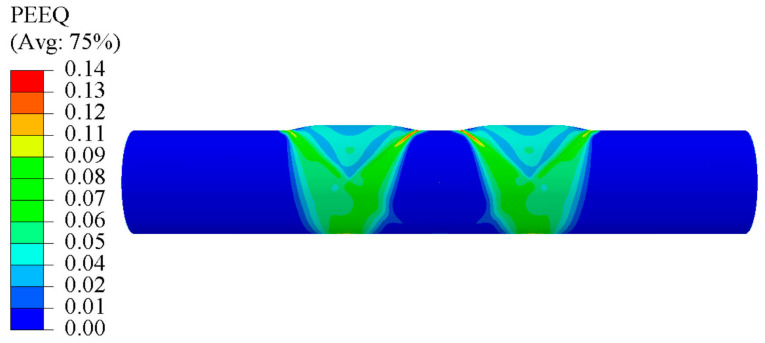
Simulation result for specimen produced via TTH process (PEEQ denotes the equivalent plastic strain).

Figure 10 shows the FEA of tube components obtained using the TTH process. Since the tube blank had the same diameter as the die cavity, there was no radial pressure on the tube components during the crushing stage. The critical deformation areas were located at the branches and the main tube under the branches. The branch height was 1.6 mm, with errors between the simulation and experiment of approximately 14.2%. The errors that occurred were caused by boundary conditions, such as lubrication during TTH, and the material properties’ inaccuracy in the numerical simulation [28]. Furthermore, the main driving force using the TTH process was the internal pressure [29]. Finally, it is worth mentioning that, as no fracture criteria were included in the simulation, the tubes did not show any fracture state.

**Figure 11:**
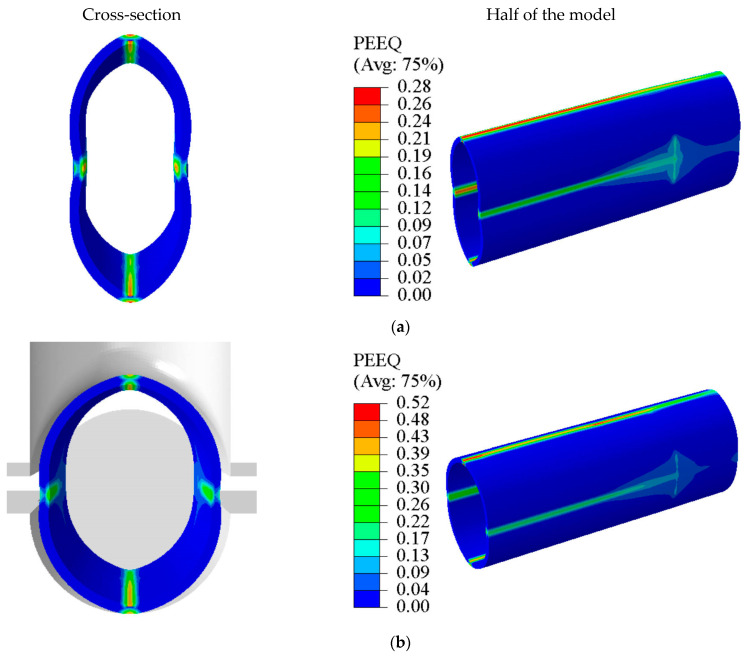

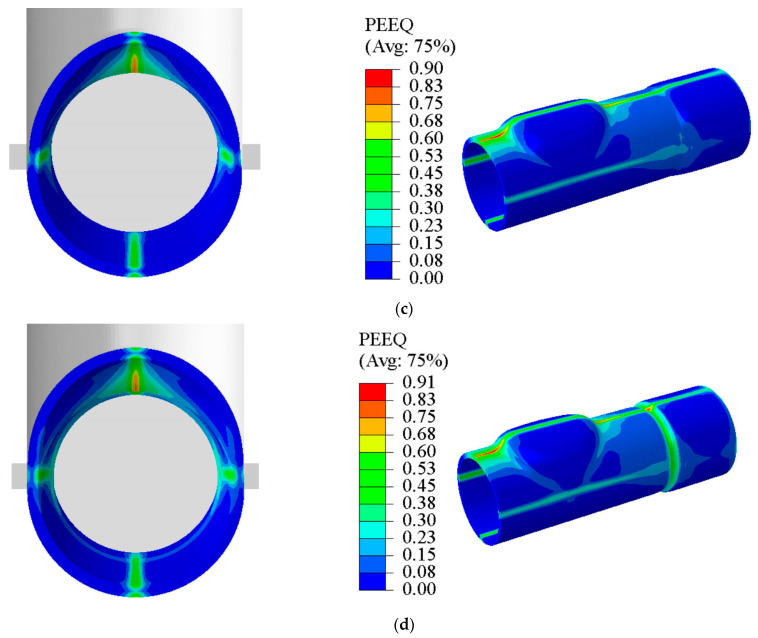
Forming of tube components using THPC process—FEA: (**a**) flattening pre-forming, (**b**) 5.8 mm crushing during the crushing stage, (**c**) 11.9 mm crushing during the crushing stage, and (**d**) hydroforming stage.

Figure 11 shows the FEA results obtained for tubes formed through the THPC. The main deformation areas were the tube top and its sides during every forming stage, which differed from the TTH process. When the tube was flattened 13 mm during the flattening stage, the straight edge of the tube blank underwent a concave deformation. Without any support inside the tube blank while flattening, a bending moment occurred when the upper plate was pressed down, resulting in a concave shape. The bending areas on both sides were curved, with a small fillet radius, as demonstrated in Figure 11a. This phenomenon was similar to the one specified in [30]. As the upper die was pressed down 5.8 mm, the concave shape of the tube blank gradually formed outward during the flattening stage under the compressive force. The tube was gradually fitted into the die cavity, as shown in Figure 11b. Hence, the flash was prevented between the upper and lower dies by the formation of a concave shape during the flattening stage. Once the upper die was pressed downward to complete the crushing stage, the material was pushed into the branch cavity using the closing force. Nevertheless, the branch had a conical cross-section shape and did not fit into the branch cavity. The branch height was 4.11 mm after crushing, as shown in Figure 11c. Finally, the branch height was 4.46 mm when the internal pressure was increased to 51 MPa, as depicted in Figure 11d. The branch was fitted into the branch die cavity as the internal pressure was increased. The branch heights obtained by simulation and experiment were 4.46 mm and 4.72 mm, respectively, i.e., a difference of 5.5%. Such results indicated that the experimental and simulation results agreed.

Therefore, the larger diameter tube was flattened. The material was subjected to radial pressure during the crushing stage, causing the material to overcome the frictional force and flow into the branch cavity. Consequently, tube formability was improved, and branch heights were increased.

**Figure 12:**
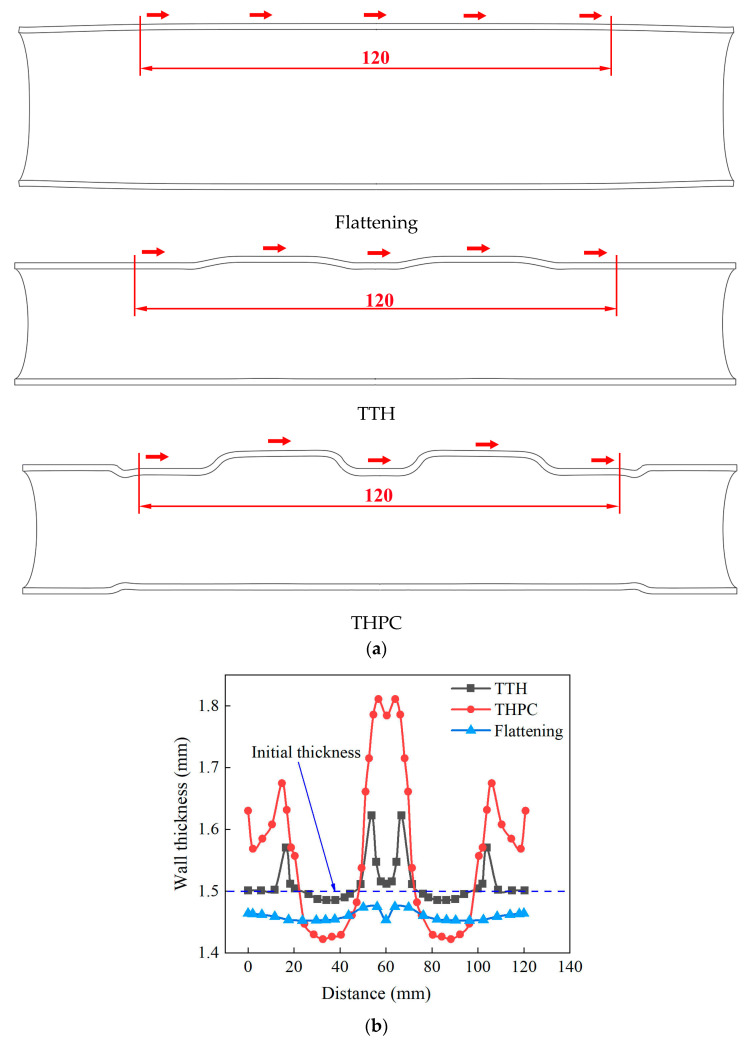
(**a**) Diagram of wall thickness measurement position (thickness measured along the direction of arrows) and (**b**) simulated wall thickness variation in the axial direction in tubes with a length of 120 mm.

Figure 12 shows the wall thickness variations in the axial direction of the tubes with a length of 120 mm as obtained through simulation. The branch’s top was the most severely thinned area in both forming processes. The minimum wall thickness of the tube formed using the TTH and THPC processes was 1.485 mm and 1.422 mm in the axial direction, respectively. Due to the limited axial feeding, the wall thickness was slightly increased at the corner between the branch and the main tube. During the flattening stage of the THPC process, the tube wall thickness was uniform, with a slight reduction. The minimum wall thickness was 1.452 mm, with a corresponding thinning rate of 3.2%. The main tube between the two branches saw an increased wall thickness of up to 20.7%. The available literature indicates minimal thickening between branches due to the absence of axial feed replenishment [7]. However, the wall thickness of the main tube between the branches was thicker in the THPC process. The main reason was that a smaller diameter tube was obtained, as the tube was compressed during the crushing stage. As per the volume invariance principle, the end wall thickness increased. 

**Figure 13:**
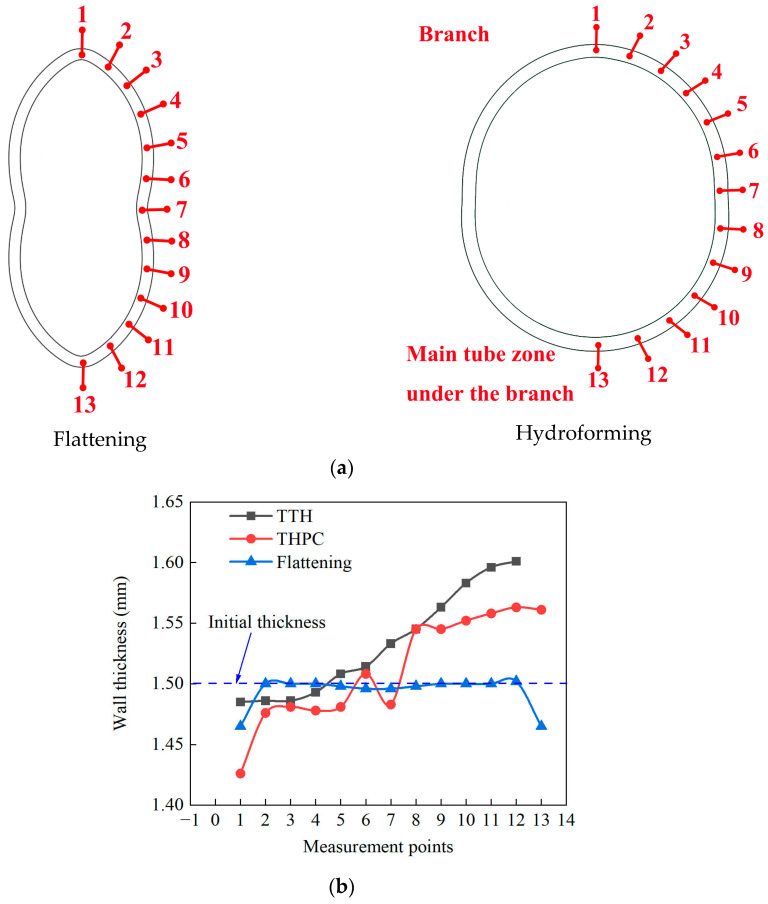
(**a**) Points for wall thickness measurement (the number show the position of the points) and (**b**) wall thickness variation of the branch in the hoop direction.

Figure 13 shows the tube wall thickness variations in the hoop direction during the flattening and hydroforming stages. The points for wall thickness measurements are shown in Figure 13a. During the flattening stage, the tube wall thickness in the hoop direction had a symmetric distribution. It was smooth, with no significant local fluctuations. The minimum thickness of 1.483 mm was located in the concave region.

The measurement points 1 to 7 were located at the branch, while points 8 to 13 were at the main tube under the branch. The wall thickness at the branch was decreased. The wall thickness at the main tube under the branch was increased during the TTH and THPC processes. The maximum tube wall thicknesses formed using the TTH and THPC processes in the hoop direction were 1.601 mm and 1.561 mm, respectively. Such findings indicated that the material flow was improved during the THPC process because the material accumulation at the main tube decreased.

**Figure 14:**
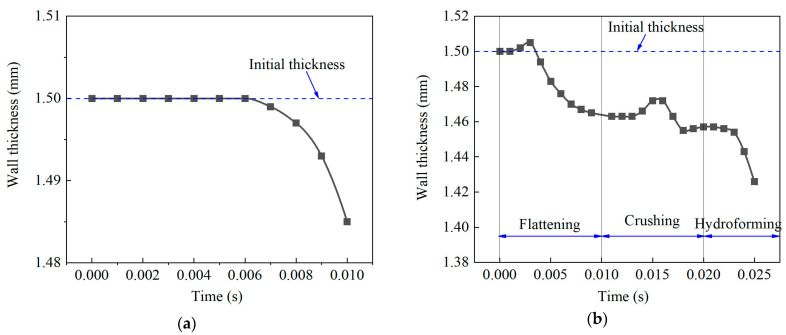
Variation of wall thickness at the top of the branch: (**a**) TTH and (**b**) THPC.

Figure 14 shows the wall thickness variation at the branch’s top during the THPC and TTH processes. During the TTH process, the wall thickness remained constant in the early forming stage due to low internal pressure and axial feeding, as shown in Figure 14a. As the internal pressure increased, the wall thickness underwent rapid thinning in the later forming stage. During the THPC process, the wall thickness was thinned from 1.5 mm to 1.465 mm at the flattening stage. The wall thickness was increased in the early crushing stage and decreased in the later crushing stage, as shown in Figure 14b. There was only a 0.009 mm decrease in wall thickness. Lastly, the variation in wall thickness was similar to that of the TTH process.

### 4.3. Stress State

**Figure 15:**
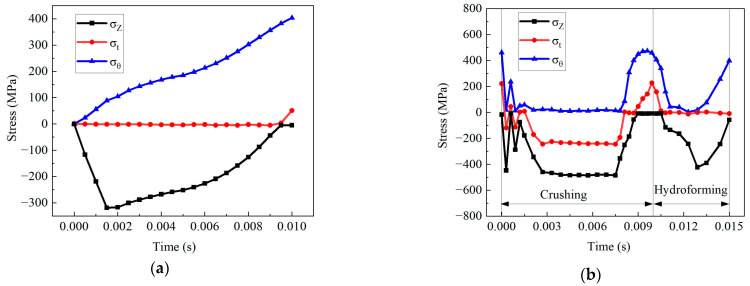
Three principal stresses at the top of the branch: (**a**) TTH and (**b**) THPC ($\sigma_{z}$ is the axial stress; $\sigma_{\theta }$ is the hoop stress; and $\sigma_{t}$ is the radial stress.).

Tube deformation was determined by its stress state [31]. Figure 15 displays the three principal stresses at the top of the branch. Figure 15a shows that the top of the branch pipe was in a plane stress state, since the radial stress was 0 MPa during the TTH process. The value of the tensile hoop stress at the top of the branch tube gradually increased, reaching a maximum value of 403.6 MPa. Due to the limited axial feeding, the axial stress of the tube was usually compressive, reaching a peak of 318.5 MPa. The compressive axial stress started to increase, then dropped, eventually coming to 0 MPa in the later stages of formation.

Figure 15b shows that the hoop stress at the top of the branch tube was 0 MPa in the early crushing stage. In contrast, the radial and axial stresses indicated compression. Following the crushing stage, the tensile hoop and the tensile radial stresses rose to 406.5 MPa and 158.6 MPa, respectively. In comparison, the axial stress approached 0 MPa. According to the wall thickness variation at the branch’s top, the thinning ratio was low when the branch height was 4.11 mm following the crushing stage. This phenomenon can most likely be attributed to the tube not being significantly affected by the friction during the crushing stage. The compressive radial and axial stresses at the early crushing stage led to a slight reduction in wall thickness. This reduction facilitated delaying the thinning of the wall during the hydroforming. 

The tube experienced significant friction as the internal pressure increased during hydroforming. The hoop stress at the top of the branch tube decreased to nearly zero before increasing to 399.5 MPa as the internal pressure increased. The axial stress was consistently compressive. The internal pressure caused the top of the branch to become thinner, ultimately causing a rupture.

**Figure 16:**
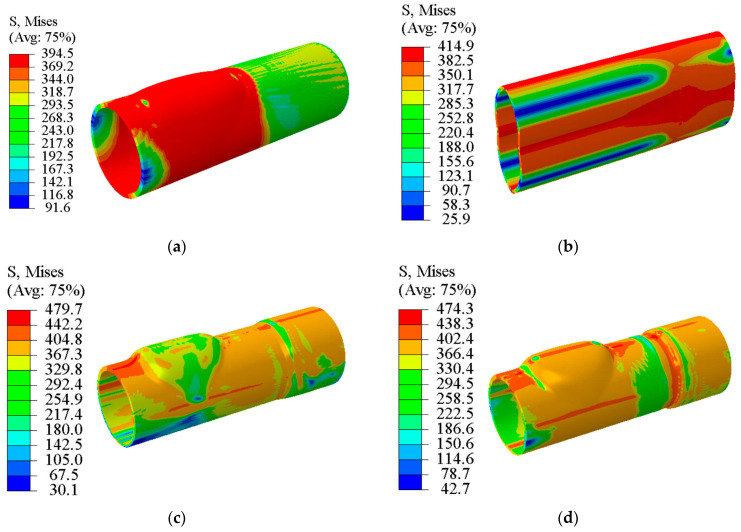
Von Mises stress contour in TTH and THPC processes (S, Mises represents effective stress): (**a**) TTH process, (**b**) flattening stage during the THPC process, (**c**) crushing stage during the THPC process, and (**d**) hydroforming stage during the THPC process.

Figure 16 shows the Von Mises stress contour in TTH and THPC processes. The highest equivalent stress in the branch area due to the high tensile stress during the TTH process was 394.5 MPa, as shown in Figure 16a. In the flattening stage during the THPC process, the highest equivalent stress reached 414.9 MPa and occurred at the top of the bending areas, as shown in Figure 16b. Following the crushing stage, as shown in Figure 16c, the highest equivalent stress in the main tube between the two branches was 479.7 MPa, indicating that the deformational areas occurred in the main tube since the tube diameter was decreased. In other words, the branch height was increased due to the reduction of the main tube diameter.

Therefore, the equivalent stress at the branch’s top was reduced to 367.1 MPa due to the bidirectional compressive stress. Similar to the TTH process, the equivalent stress at the top of the branches increased because of the high internal pressure, as shown in Figure 16d. The equivalent stress increased on both branches due to friction when the internal pressure increased. Therefore, the flattening, pre-forming, and crushing stages caused work hardening, strengthening the branch top and preventing rapid thinning, eventually causing cracking.

## 5. Conclusions

This article proposed a new THPC process for fabricating a parallel double tube. The experiments and numerical simulations were used to analyze the impact of the TTH and THPC on the forming performance of parallel double-branch tubes. The following conclusions were drawn:

(1) The branch height can be significantly increased when using the THPC process. In comparison to the TTH, the branch height was increased 2.37-fold.
(2) In the THPC process, a tube blank with a larger diameter was used to provide additional material to form the parallel double tube. The larger diameter tube was flattened for pre-forming. The tube was subjected to radial pressure during the crushing stage to overcome the friction. Additional material could be pushed into the branch cavity. Moreover, the top of the branch was subjected to bidirectional compressive stress. It was found that such a stress state can delay the reduction in wall thickness, effectively enhancing the specimen formability performance.
(3) The flattening, pre-forming, and crushing stages resulted in the specimen’s work hardening when using the proposed THPC process. The ability to resist rupture during the hydroforming stage was significantly improved by altering the stress state of the tube.

The effect of the tube blank flattening magnitude and loading path at the crushing and hydroforming stages on the forming performance of parallel double-branch tubes will be investigated in future work.

## Figures and Tables

**Table 1:** Experimental schemes of observed forming processes.

Forming Process	Process Stages	Process Parameters	Values
TTH	Axial feeding stage	Feeding amount	1.5 mm
	Hydroforming stage	Burst internal pressure	40 MPa
THPC	Flattening stage	Flattening amount	13 mm
	Crushing stage	Internal pressure	0 MPa
	Axial feeding stage	Feeding amount	1.5 mm
	Hydroforming stage	Burst internal pressure	51 MPa

## Data Availability

Data will be available upon request through the corresponding author.
